# Nuclear Magnetic Resonance Reveals Molecular Species in Carbon Nanodot Samples Disclosing Flaws

**DOI:** 10.1002/anie.202200038

**Published:** 2022-02-21

**Authors:** Beatrice Bartolomei, Andrea Bogo, Francesco Amato, Giulio Ragazzon, Maurizio Prato

**Affiliations:** ^1^ Department of Chemical and Pharmaceutical Sciences INSTM UdR Trieste University of Trieste via Licio Giorgieri 1 34127 Trieste Italy; ^2^ Institut de Science et d'Ingénierie Supramoléculaires UMR7006 University of Strasbourg, CNRS 8 allée Gaspard Monge 67000 Strasbourg France; ^3^ Centre for Cooperative Research in Biomaterials (CIC BiomaGUNE) Basque Research and Technology Alliance (BRTA) Paseo de Miramón 194 20014 Donostia San Sebastián Spain; ^4^ Basque Fdn Sci Ikerbasque 48013 Bilbao Spain

**Keywords:** Carbon Dots, Chirality, Molecular Species, Nuclear Magnetic Resonance, Optical Properties

## Abstract

Carbon nanodots are currently one of the hot topics in the nanomaterials world, due to their accessible synthesis and promising features. However, the purification of these materials is still a critical aspect, especially for syntheses involving molecular precursors. Indeed, the presence of unreacted species or small organic molecules formed during solvothermal treatments can affect the properties of the synthesized nanomaterials. To illustrate the extreme importance of this issue, we present two case studies in which insufficient purification results in misleading conclusions regarding the chiral and fluorescent properties of the investigated materials. Key to identify molecular species is the use of nuclear magnetic resonance, which proves to be an effective tool. Our work highlights the need to include nuclear magnetic resonance as a standard characterization technique for carbon‐based nanomaterials, to minimize the risk of observing properties that arise from molecular species, rather than the target carbon nanodots.

## Graphical abstract

The purification of carbon nanodots is a critical aspect in their preparation, especially for syntheses involving molecular precursors. Indeed, the presence of organic molecules in the final sample can lead to mistaken conclusions regarding their properties. In this work, NMR is proven to be an essential technique to verify the purification of carbon nanodots, thanks to its ability to disclose molecular species.

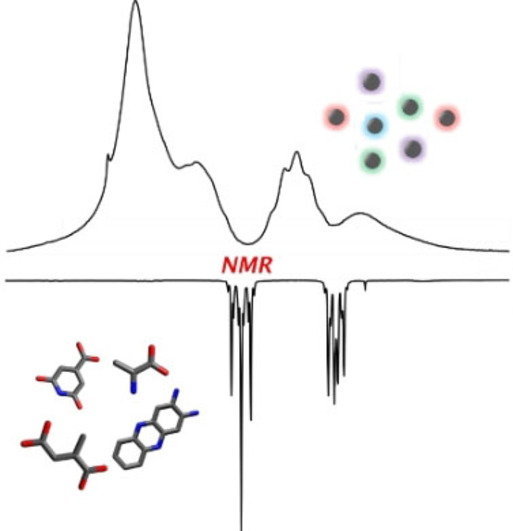

Purification has played a crucial role in the field of carbon nanodots (CNDs) since their discovery. They were found accidentally in 2004 in the purification of single wall carbon nanotubes.^[1]^ From then on, CNDs have become one of the most studied carbon‐based materials, owing to their promising properties, such as their fascinating photoluminescence, as well as their apparently easy preparation.[^2^, ^3^, ^4^, ^5^] Generally, CNDs are synthesized following either top‐down or bottom‐up approaches, with the latter more commonly used, due to their superior versatility and accessibility.^[6]^ In particular, the solvothermal treatment of small organic molecules has become a very important production process for this type of nanoparticles.[^7^, ^8^, ^9^] Nevertheless, the high temperature treatment of the precursors enables multiple reaction pathways resulting in the formation of CNDs together with small molecular weight side‐products. The effective removal of these species from the crude product can be challenging.[^10^, ^11^] In case of an incomplete purification, the presence of these species in the final sample can influence the observed features, resulting in the attribution of the properties of the molecular species to CNDs. As an example, molecular fluorophores were identified as responsible for the observed photoluminescence properties of certain CNDs.[^12^, ^13^, ^14^, ^15^, ^16^] Despite this critical point being frequently highlighted, insufficient purification remains widespread in the literature, preventing the maturation of the field and making it difficult to understand the fundamental properties of these emerging nanoparticles.

One of the reasons why the purification problem is so widespread is that the commonly employed characterization techniques, such as optical spectroscopies, X‐ray photoelectron spectroscopy (XPS), atomic force microscopy (AFM), and transmission electron microscopy (TEM) are not able to reveal the presence of molecular species in CND samples. As an example, AFM and TEM are not able to differentiate between carbon nanoparticles and aggregates of organic molecules. Indeed, as reported in the literature, CND‐like structures with a d‐spacings of 0.21 nm can be observed performing measurements on pure molecular samples.[^17^, ^18^] Thus, even when particles are observed, this piece of evidence does not rule out the presence of molecular species. Therefore, complementary techniques are needed to confirm the effective purification.

A possible strategy to confirm effective purification is to employ the key characterization technique of organic chemistry, namely nuclear magnetic resonance (NMR). Indeed, the expected signals for CNDs are very different from those of simple organic molecules. The ^1^H NMR spectrum of CNDs should not present sharp and resolved signals, as a result of their complex and extremely heterogeneous nature. Protons experience different environments resulting in broad and non‐resolved peaks, similar to those usually reported for polydispersed polymers. On the contrary, small molecules typically display sharp signals, which should make it possible to identify their residual presence in CND samples. In principle, if NMR shows the presence of sharp peaks, one should go back to the purification steps, until only broad signals are observed (Figure 1).

**Figure 1:**
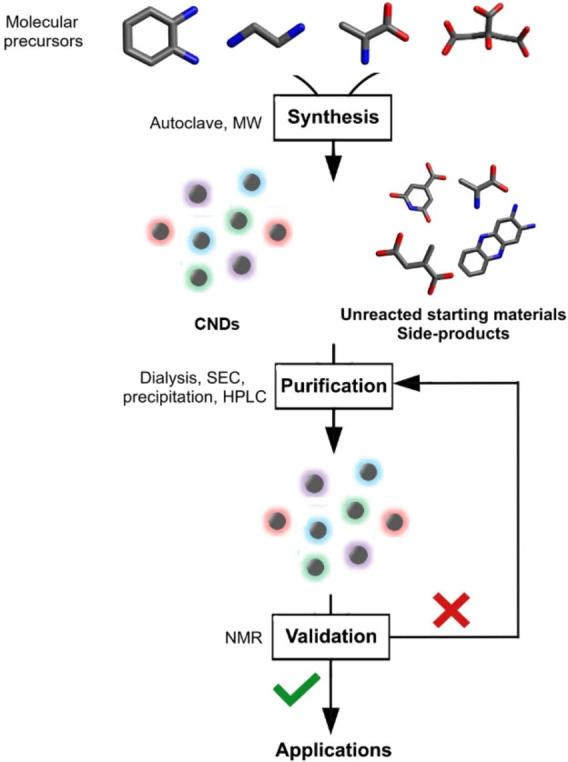
Flow chart associated to the synthesis, purification, and validation of a CND sample. MW=microwave; SEC=size exclusion chromatography.

To illustrate the effectiveness of ^1^H NMR as useful characterization technique, we have investigated two case studies, selecting CNDs that are obtained from widely used precursors. In particular, in line with the latest developments of the field, we have selected CNDs displaying chiral properties and fluorescence in the red region of the spectrum.[^19^, ^20^] These are, in fact, key properties to push forward the applications of CNDs in many fields, ranging from sensing to catalysis.[^21^, ^22^, ^23^, ^24^, ^25^, ^26^, ^27^] Strikingly, a simple, fast and straightforward NMR analysis may often reveal that *the properties of interest arise from molecular species present as impurities rather than from CNDs*.

To demonstrate that even minimal variations to consolidated protocols require careful verification of effective purification, as a first case study we focused on a versatile protocol for the bottom‐up synthesis of CNDs starting from arginine (Arg) and ethylenediamine.[^28^, ^29^] The versatility of this synthetic protocol was demonstrated in several occasions by our group.^[30]^ Indeed the protocol supports a variety of doping agents and changes in the diamine precursor, making it possible to modulate the properties of CNDs such as their fluorescence, band gap, and chirality.[^31^, ^32^, ^33^, ^34^] Here, the preparation of chiral CNDs was attempted by using the same synthetic protocol, while replacing ethylenediamine with *(R,R)‐*1,2‐diphenylethylenediamine (*RR*‐Dphen) or its (*S*,*S*)‐ enantiomer as precursors (Figure 2a). In line with analogous syntheses, in the process of microwave heating, the precursors mixture changed from colorless to dark brown (Figure S1). After filtration, the solution was dialyzed against milli‐Q water for 48 hours, obtaining a solid sample after freeze drying, defined as *RR*‐ or *SS*‐CNDs‐**1** depending on the Dphen enantiomer employed.

**Figure 2:**
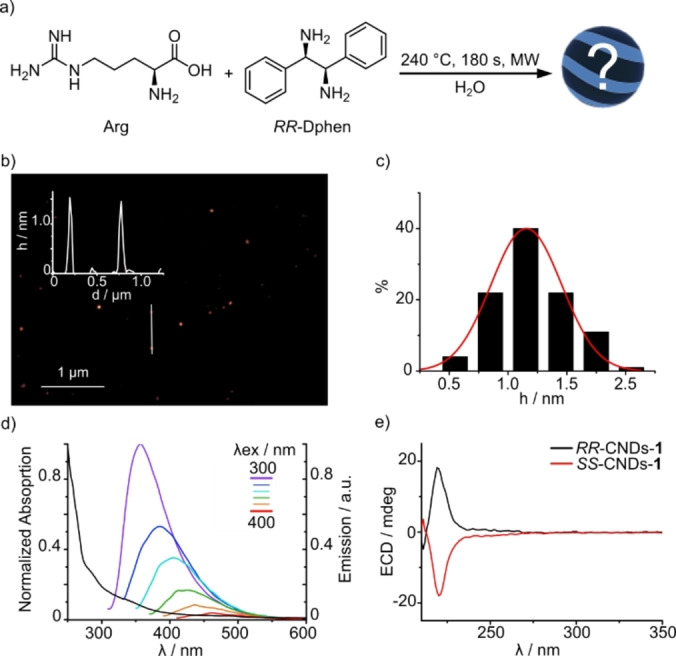
a) Reaction scheme for the synthesis of *RR*‐CNDs‐**1**. b) Tapping mode AFM of *RR*‐CNDs‐**1** drop‐cast on a mica substrate from an aqueous solution; inset is the height profile along the white line. c) Size histogram of AFM height data, with distribution fit (red curve) based on a Gaussian distribution. d) UV/Vis absorption and emission spectra of *RR*‐CNDs‐**1** recorded at different excitation wavelengths in water. e) ECD spectrum of *RR*‐CNDs‐**1** (black line) and *SS*‐CNDs‐**1** (red line) recorded in water.

The characterization of CNDs‐**1** was in line with the expectations. The morphological characterization was performed using AFM, which confirmed the presence of round shape nanoparticles with an average size of 1.2± 0.3 nm (Figure 2b, c). The UV/Vis absorption spectrum of *RR*‐CNDs‐**1** presents a broad absorption with a non‐structured shoulder at around 280 nm (Figure 2d). The emission spectra show a main emission band centered at 350 nm and an excitation wavelength‐dependent profile. Accordingly, the luminescence quantum yield changes with the excitation wavelength, with a value of 7 % at 350 nm (Figure S2). Thus, the absorption and emission properties resemble those of typical CND samples.[^28^, ^29^] Similar results were observed for *SS*‐CNDs‐**1** (Figures S3, S4). To investigate the chiral nature of the synthesized CNDs‐**1** the electronic circular dichroism (ECD) spectra were recorded. The ECD spectrum of *RR*‐CNDs‐**1** aqueous solution presents a positive Cotton effect at 220 nm (Figure 2e). This spectroscopic evidence confirms that the chirality of *RR*‐Dphen has been transferred to *RR*‐CNDs‐**1**, because Arg is known to racemize under the synthesis conditions.^[31]^ In the case of *SS*‐CNDs‐**1**, a mirror image of the ECD signal was observed (Figure 2e).

The characterization reported so far is the classical one for CNDs, being in this case *apparently* coherent with the successful preparation of chiral, blue‐emissive CNDs. To investigate the presence of molecular impurities in CNDs‐**1**, NMR was employed. The ^1^H NMR spectrum of *RR*‐CNDs‐**1** displays broad signals in the aliphatic region (1–4 ppm) while intense sharp signals can be seen in the aromatic region (7–7.5 ppm) and at 5 ppm (Figure 3a). The presence of sharp signals points at the presence of molecular species, what is confirmed by comparing the ^1^H NMR spectrum of *RR*‐CNDs‐**1** with that of *RR*‐Dphen, which presents exactly the same signals (Figure 3b). Analogous results are obtained for *SS*‐CNDs‐**1** (Figure S5). These spectra demonstrate that Dphen is still present in the final product and this piece of evidence throw doubts on the origin of the observed ECD signals.

**Figure 3:**
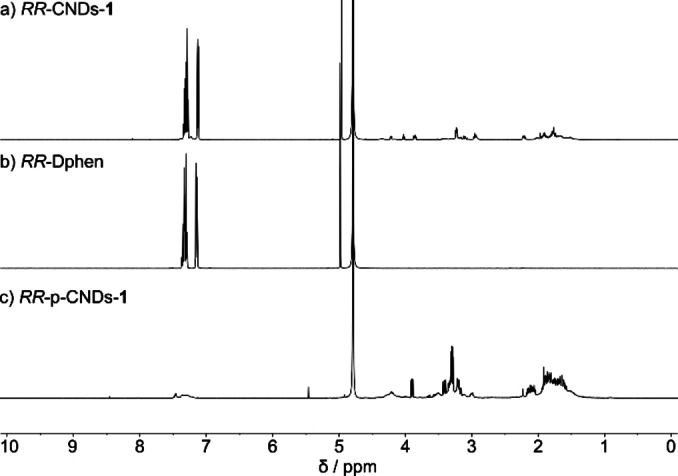
^1^H NMR spectra (D_2_O/Trifluoroacetic acid (TFA) 3 % v/v, 400 MHz, r.t.) of a) *RR*‐CNDs‐**1**, b) *RR*‐Dphen, c) *RR*‐p‐CNDs‐**1**.

To clarify the presence of Dphen after the dialysis, a control experiment was performed. A saturated solution of *RR*‐Dphen was prepared and treated with the same purification protocol used for CNDs. After filtration and dialysis for 48 hours, *RR*‐Dphen was still present inside the dialysis bag with a mass percentage of around 8 % with respect to the saturated solution. This result shows how purification protocols should be carefully validated, even when minimal modifications are studied.^[35]^ Caution should be taken especially when using organic precursors sparingly soluble in water: dialysis in water may not work in these cases.

The ^1^H NMR spectrum of *RR*‐CNDs‐**1** indicates the need to further purify the sample from Dphen, which could be successfully achieved by performing a liquid‐liquid extraction using dichloromethane and water. The ^1^H NMR spectrum of the purified *RR*‐CNDs (*RR*‐p‐CNDs‐**1**) shows successful removal of Dphen, indicated by the complete disappearance of the sharp peaks at 7–7.5 ppm and 5 ppm (Figure 3c). Importantly, the removal of Dphen from *RR*‐CNDs‐**1** is reflected by the absence of any ECD signal, demonstrating that the chiral properties observed above for *RR*‐CNDs‐**1** derive exclusively from the unreactive chiral precursor (Figure S6a). The presence of relatively sharp peaks in the aliphatic region of the spectrum of *RR*‐p‐CNDs‐**1** suggests the presence of yet other molecular components, possibly due to self‐condensation of Arg.^[36]^ Work along the identification of these species is currently underway in our laboratories. On the other hand, the absorption and fluorescence properties remained essentially unchanged after the additional purification step, corroborating the idea that indeed the observed luminescence properties arise from carbon particles which are still observable under AFM (Figure S6b, c).

To illustrate that an ineffective purification can impact multiple properties at the same time, we selected a second case study where chiral and red‐emissive polymer dots were claimed to display circularly polarized emission when embedded in a suitable chiral matrix.^[37]^ According to the reported protocol, the synthesis was performed treating *o‐*phenylendiamine (*o*‐PDA) and L*‐*tryptophan (Trp) at 160 °C in an acidic environment and purifying the sample through dialysis (Figure 4a). The successful reproduction of the synthetic protocol was demonstrated employing absorption, emission, and ECD spectroscopies, which indicated the preparation of a chiral material with emission centered at 600 nm, termed CNDs‐**2** (Figure S8). The morphological characterization of CNDs‐**2** was performed using AFM that confirmed the presence of nanoparticles with an average size of 4± 1 nm (Figure S9).

**Figure 4:**
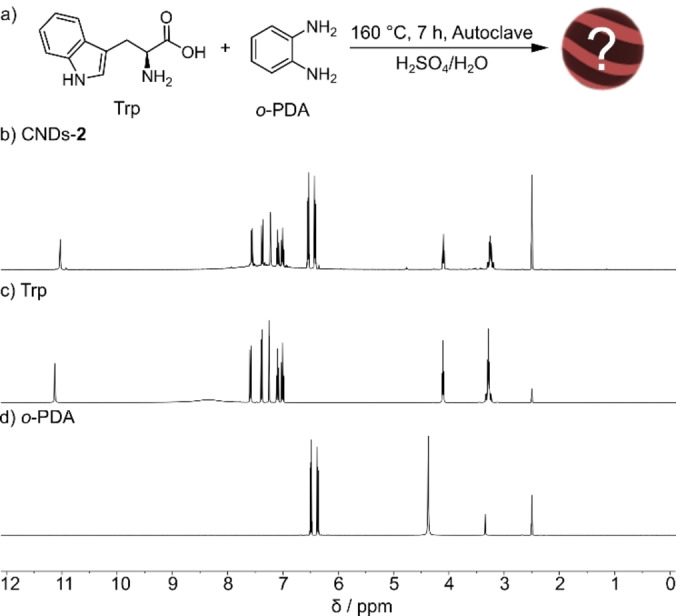
a) Reaction scheme for the synthesis of CNDs‐**2**. b–d) ^1^H NMR spectra (DMSO‐d_6_, 400 MHz, r.t.) of CNDs‐**2**, Trp with 1 eq. of TFA‐d, and *o‐*PDA, respectively.

To check the absence of molecular species in CNDs‐**2**, the ^1^H NMR spectrum was recorded (Figure 4b). The observed signals are sharp, coincide with those reported in ref. [37] and fit quite well those of the starting materials (Figure 4c, d). In particular, in the region between 6–8 ppm the aromatic pattern of both Trp and *o*‐PDA can be observed. This result demonstrates that dialysis in water is not a suitable technique to purify this mixture, which can be reasonably attributed to the limited solubility of the species in water. Indeed, a precipitate is formed inside the dialysis bag during the purification process, preventing the effective removal of molecular species even when their molecular weight is less than the cut off of the membrane.

Prompted by the NMR evidence, high‐performance liquid chromatography (HPLC) was employed to fully separate the unreacted species from the final products. The chromatogram was monitored at two wavelengths, namely 254 and 575 nm (Figure 5a and S11, respectively). The trace at 254 nm reveals four distinct fractions (#1–#4), of which only fraction #4 presents a significative absorption at 575 nm. Considering the mass recovered after semipreparative HPLC separation, fraction #1 accounts for 43 %, #2 for 45 %, #3 for 7 % and #4 for just 4 % of the injected sample, summing up to a total of 99 % mass recovered. We have characterized all these fractions employing absorption, emission, ECD, and NMR spectroscopies, as well as mass spectrometry.

**Figure 5:**
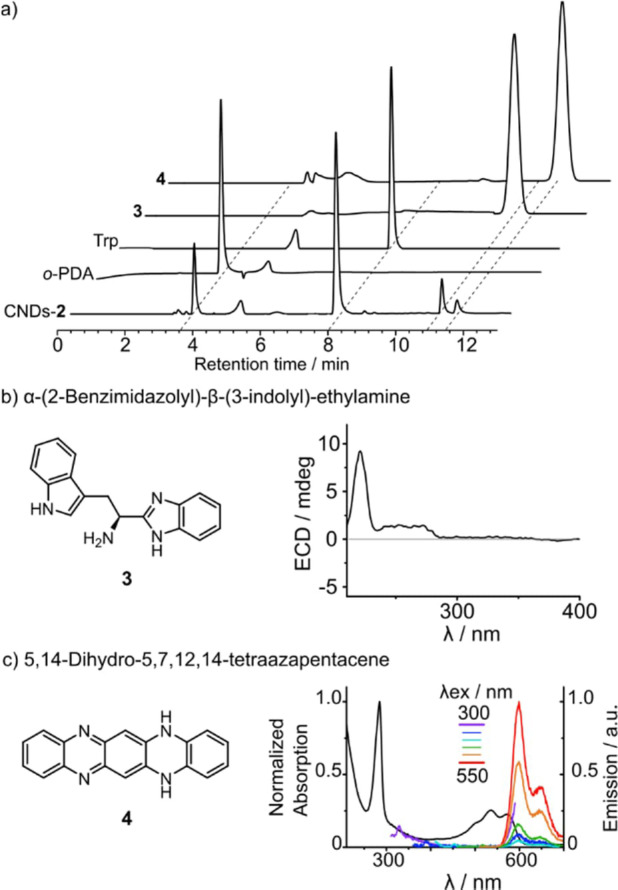
a) Analytical C8‐HPLC traces of CNDs‐**2** and, for comparison, those of commercial *o*‐PDA, commercial Trp, synthesized compound **3**, and synthesized compound **4**, monitored at 254 nm; signals in the HPLC trace observed at ca. 5 min arise from *N,N*‐dimethylformamide used as the injection solvent. b) Molecular structure of synthesized compound **3** and its ECD spectrum in ethanol solution. c) Molecular structure of synthesized compound **4**, its absorption and emission spectra recorded at different excitation wavelengths in ethanol solution.

Fractions #1 and #2 present a blue emission and a positive ECD signal which is very similar to that reported for pure Trp (Figures S12–S17). The main ^1^H NMR signals of these fractions are due to *o*‐PDA and Trp. Their presence is corroborated by mass spectrometry data and the identical elution time of pure *o*‐PDA and Trp with respect to fraction #1 and #2, respectively.

Fraction #3 is characterized by a blue emission and a typical ECD signal (Figure S18). The ^1^H NMR signals confirm the presence of a derivative containing Trp and *o‐*PDA moieties, while the mass spectrum reveals a species with a m/z of 277 (Figures S19 and S20). Considering the experimental conditions employed for the synthesis of CNDs‐**2**, we have identified α‐(2‐benzimidazolyl)‐β‐(3‐indolyl)‐ethylamine (**3**) as a possible candidate (Figure 5b). The synthesis of this compound was performed according to a reported protocol,^[38]^ followed by HPLC purification. Compound **3** presents the same elution time as fraction #3, and evidence of structural identity between these two compounds was provided by NMR and mass spectrometry (Figures S21 and S22). The isolated molecule shows a blue emission joined by a positive ECD signal, which are compatible with that reported for fraction #3, further supporting the hypothesis that **3** is the principal component of this fraction (Figure 5b and S23).

Fraction #4 presents an emission band centered at 600 nm, but no ECD signals (Figure S24). ^1^H NMR presents broad signals in the aromatic region and a sharp singlet around 6 ppm. The addition of triethylamine to the NMR solution leads to the formation of resolved signals (Figure S25). This result suggests that in acid solution we might be in the presence of aggregates, which are then broken in basic environment. From the mass spectrum, a species with a *m*/*z* of 285 is revealed (Figure S26). 5,14‐Dihydro‐5,7,12,14‐tetraazapentacene (**4**) (Figure 5c) was identified as a possible by‐product originating from *o*‐PDA. Following a reported procedure,^[39]^ we synthesized compound **4**, to compare its properties with fraction #4. Data obtained using ^1^H NMR, absorption and emission spectroscopies, as well as mass spectrometry, perfectly match the characterization reported for fraction #4 (Figure 5c and S27 and S28).

The further HPLC purification performed for CNDs‐**2** cast heavy doubts on the results claimed in ref. 37. Basically, the fluorescent properties have to be attributed to compound **4** rather than to CNDs‐**2**. Also, the chiroptical properties are assigned to residual Trp and compound **3** and not to CNDs‐**2**. Moreover, AFM measurements cannot be considered a sufficient proof to demonstrate the presence of CNDs. Indeed, even the simple deposition of pure solution of Trp on a mica substrate allows to observe nanoparticles which are, in reality, molecular aggregates (Figure S30).^[40]^ The reported CPL is therefore most probably due to compound **4** embedded in the chiral polymer matrix. In fact, it is known that achiral fluorescent species, incorporated in appropriate chiral matrices, display CPL.[^41^, ^42^]

Taken together, these case studies illustrate that the high temperature treatment involved in solvothermal synthesis is not always coupled with a carbonization of the precursors, especially when aromatic molecules are used as starting materials. The production of organic by‐products may strongly affect the properties of the final sample as clearly demonstrated in the examples reported in this paper. The use of NMR is therefore suggested as a useful analytical technique to investigate the presence of molecular components in CND samples.

In summary, in this work we have demonstrated the critical role of NMR spectroscopy in the characterization of CNDs. We have investigated two case studies for the synthesis of chiral emissive CNDs. In the first case, CNDs were prepared using a consolidated synthetic protocol employing Dphen as the chiral dopant. Recording the ^1^H NMR spectrum of the final sample was essential to reveal the presence of the starting diamine. A further purification procedure assessed that unreacted Dphen is responsible for the chiroptical properties in a first step associated to CNDs (Figure 6a). In the second case study, chiral red emissive CNDs were synthesized employing *o‐*PDA and Trp as starting materials. Also in this case, the ^1^H NMR spectrum revealed the presence of molecular species in the synthesized sample. An additional purification step was performed using HPLC, which showed that the red‐emission and the chiral properties come from molecular species present in the sample. In particular the red‐emission can be attributed to aromatic molecule **4**, while the chiral properties are related to unreacted Trp and its derivative **3** (Figure 6b).

**Figure 6:**
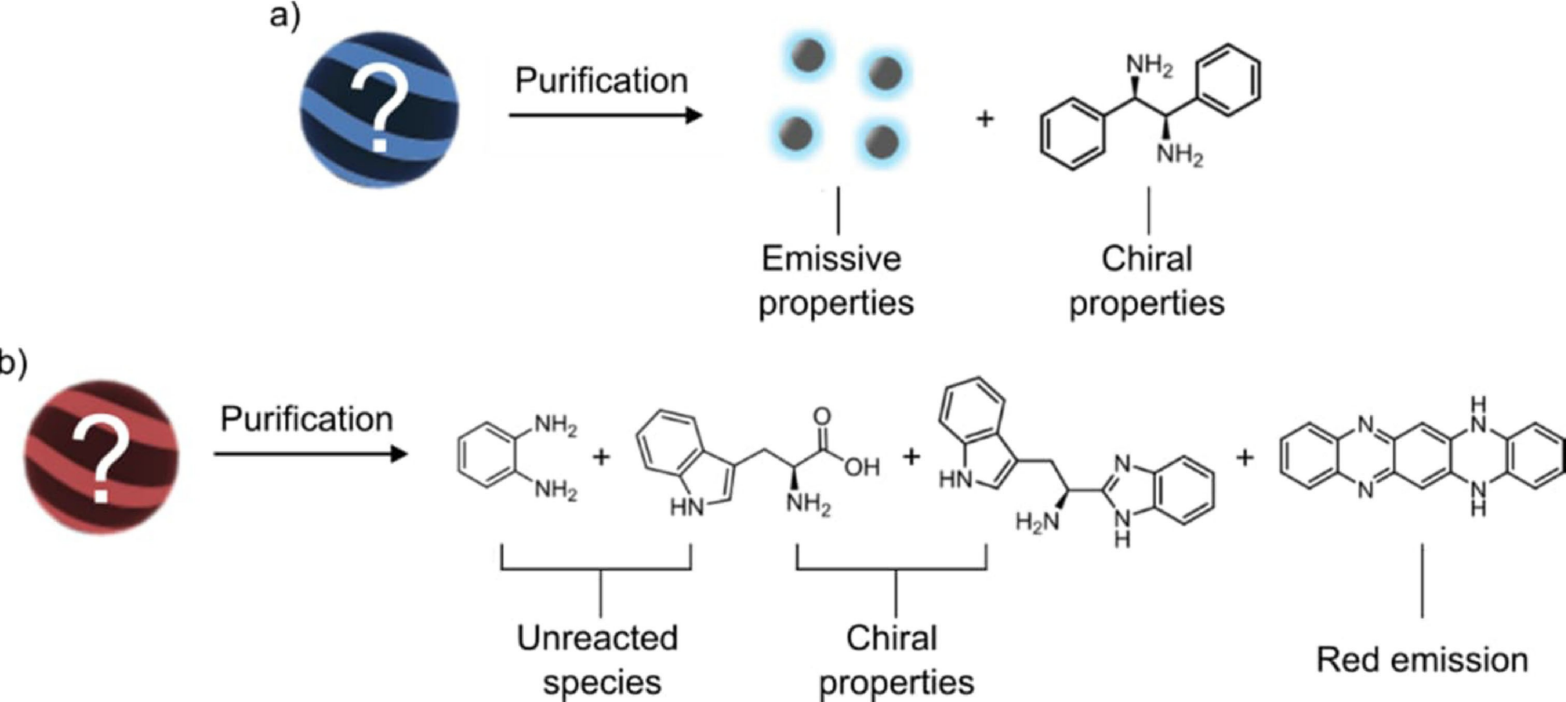
Structure and properties of species identified after the purification of a) CNDs‐**1**  and b) CNDs‐**2** .

The results presented here indicate that inconsistencies and errors in published work may be widespread and in need of re‐evaluation. Indeed, the elusive nature of CNDs points at the necessity of thoroughly testing the proposed conclusions. To this aim, NMR can prove to be an essential technique to minimize the risk of residual molecular species in the final material, since it can reveal the presence of molecular species in a fast and easy‐operative way.

## Conflict of interest

The authors declare no conflict of interest.

## Supporting information

As a service to our authors and readers, this journal provides supporting information supplied by the authors. Such materials are peer reviewed and may be re‐organized for online delivery, but are not copy‐edited or typeset. Technical support issues arising from supporting information (other than missing files) should be addressed to the authors.

Supporting Information

## Data Availability

The data that support the findings of this study are available in the supplementary material of this article.
