# Supplementary material for: Nuclear Magnetic Resonance Reveals Molecular Species in Carbon Nanodot Samples Disclosing Flaws
Source: Angew Chem Int Ed Engl. 2022 Feb 21;61(20):e202200038. doi: 10.1002/anie.202200038 (PMC9304307; doi:10.1002/anie.202200038)
Supplement: Supplementary file 1 — Supporting Information [file ANIE-61-0-s001.pdf]

Angewandte Chemie
Eine Zeitschrift der Gesellschaft Deutscher Chemiker

Supporting Information

Nuclear Magnetic Resonance Reveals Molecular Species in Carbon Nanodot Samples Disclosing Flaws

*B. Bartolomei, A. Bogo, F. Amato, G. Ragazzon, M. Prato**

Table of Contents

A. General information 4

B. CNDs-**1** 5

B1. Synthesis 5

B2. Characterization of *RR*-CNDs-**1** 5

B3. Characterization of *SS*-CNDs-**1** 6

B4. Characterization of *RR*-p-CNDs-**1** 7

B5. Characterization of *RR*-Dphen 7

C. CNDs-**2** 8

C1. Synthesis 8

C2. Characterization 8

C3. Fraction #1 10

C4. Fraction #2 12

C5. Fraction #3 13

C6. α-(2-Benzimidazolyl)-β-(3-indolyl)-ethylamine (**3**) 15

C6.1 Synthesis 15

C6.2 Characterization 15

C7. Fraction #4 17

C8. 5,14-dihydro-5,7,12,14-tetraazapentacene (**4**) 19

C8.1. Synthesis 19

C8.2 Characterization 19

C9. Characterization of *L*-Tryptophan 21

D. References 22

A. General information

Synthesis. Chemicals were purchased from Sigma Aldrich, TCI, Alfa Aesar and Fluorochem and were used as received unless otherwise stated. Solvents were purchased from Sigma Aldrich and Alfa Aesar, while deuterated solvents from Eurisotop and Sigma Aldrich. Ultrapure fresh water obtained from a Millipore water purification system (>18MΩ Milli-Q, Millipore) was used in all experiments. Microwave synthesis was performed on a CEM Discover-SP, using 10 mL glass microwave vials. For filtration, Merck Omnipore 0.1 µm PTFE Membrane filters (25 mm) were employed. Solutions were concentrated under reduced pressure on a Büchi rotatory evaporator or freeze-dried using CoolSafe 9L; Labogene, model no. CoolSafe-9 lyophilizer. ***Purification.*** Dialysis tubes with molecular weight cutoff 0.5-1 kDa were bought from Spectrum Labs. For thin layer chromatography (TLC) analysis, Merck pre-coated TLC plates (silica gel 60 GF254, 0.25 mm) were employed, using UV light as the visualizing agent (254 nm, 365 nm). Analytical HPLC separation was carried out employing Agilent Infinity II. The UV-Vis chromatograms were monitored at 254 nm and 575 nm. A Phenomenex C8 column (150x4.6 mm) with 3 um pore size was used for HPLC separation. The injection volume was 5 uL, and the column temperature was maintained at 40 °C. The mobile phase was prepared using binary mixtures of acetonitrile and water (containing 1% of formic acid). The flow rate of the mobile phase was set at 0.5 mL/min. The eluted gradient was programmed as the percentage of acetonitrile was linearly increased from 0 to 100% in 45 minutes. Semipreparative HPLC separation was carried out employing Agilent 1260 Infinity. A Phenomenex C18 column (250x10 mm) with 5 um pore size was used for HPLC separation. The injection volume was 500 uL, and the column temperature was maintained at 40 °C. The mobile phase was prepared using binary mixtures of acetonitrile and water (containing 0.05% of trifluoroacetic acid). The flow rate of the mobile phase was set at 3 mL/min. The eluted gradient was programmed as the percentage of acetonitrile was linearly increased from 20 to 100% in 45 minutes. ***Characterization.*** UV-Vis measurements were carried out on Varian Cary 5000 spectrophotometer, produced by Agilent. The fluorescence spectra were recorded on Varian Cary Eclipse spectrofluorimeter, while the electronic circular dichroism (ECD) spectra were recorded on Jasco J-810 spectrophotometer. All the measurements were performed at room temperature (20°C) with 10 mm path length quarz cuvettes. ECD conditions were as follows: scanning rate 20 nm/min, data pitch 1 nm, Digital Integration Time (D.I.T.) 8 s, 6 accumulations. Quantum yield (QY) measurements were performed with quinine sulphate in 0.10 M H_2SO_4 (literature quantum yield 0.54 at 360 nm)[1] as standard and CNDs-**1** in aqueous solution. The fluorescence quantum yields were calculated according to the following equation: $\Phi x = \Phi_{st}(I_x A_{st} n_x^2)/(I_{st} A_x n_{st}^2)$, I is the measured integrated fluorescence emission intensity. A is the absorbance value, n is the refractive index of the solvent and Φ is the quantum yield. The index x is referred to the sample, and the index st is referred to the standard. The absorbance of the standard and the sample were measured with A<0.1 at the excitation wavelength. The quantum yield was calculated at different excitation wavelengths by taking advantage of the following proportion: $\Phi_{exc} \propto I_{exc}/A_{exc}$.Where the subscript exc indicates a specific excitation wavelength. The estimated experimental error on QY is 25%. AFM images were obtained with a Nanoscope IIIa, VEECO Instruments. As a general procedure to perform AFM analyses, tapping mode with a HQ:NSC19/ALBS probe (80kHz; 0.6 N/m) (MikroMasch) from drop cast of samples in an aqueous or methanol solution (concentration in the order of µg/mL) on a mica substrate was performed. The obtained AFM-images were analyzed in S3 Gwyddion 2.35. Varian Inova 400 MHz was used to record the ^{1}H-NMR spectra. Low resolution mass spectra (LRMS) were obtained on Bruker Esquire 4000 (ESI). High resolution mass spectra (HRMS) were obtained on Bruker micrOTOF-Q (ESI-TOF).

B. CNDs-1

B1. Synthesis

RR-CNDs-**1** and *SS*-CNDs-**1** were obtained via microwave irradiation of an aqueous solution of *L*-Arginine (Arg) and (*R,R*)-(+)-1,2-Diphenylethylenediamine or (*S,S*)-(-)-1,2-Diphenylethylenediamine (Dphen). Typically, Arg (87.0 mg), Dphen (106.1 mg) and Milli-Q water (100.0 µL) were heated at 240 °C, 100 psi and 200 W for 180 seconds. In the process of microwave heating, the solution changed from colorless to dark brown (Figure S1). The reaction mixture was then diluted with water and the solution was filtered through a 0.1 µm microporous membrane separating a slightly yellow solution that was dialyzed against pure water through a dialysis membrane (cut off 500-1000 Da) for 2 days. The aqueous solution was then lyophilized giving a yellow solid (*RR*-CNDs-**1**: 50.3 mg; *SS*-CNDs-**1**: 52.2 mg).

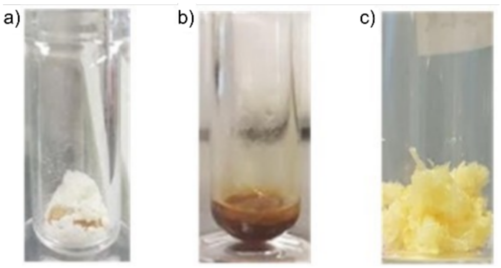

Figure S1. Photographs of the solution before (a) and after (b) the heating process. c) CNDs-**1** after lyophilization.

*Purification of CNDs-**1**:* *RR*-CNDs-**1** and *SS*-CNDs-**1** were further purified with a liquid-liquid extraction. The CNDs-**1** were dissolved in Milli-Q water (15 mL) and extracted seven times with CH_2Cl_2 (3 mL). The effective removal of the Dphen was confirmed by TLC of the organic phase and recording ^{1}H-NMR of the aqueous solution.

B2. Characterization of *RR*-CNDs-1

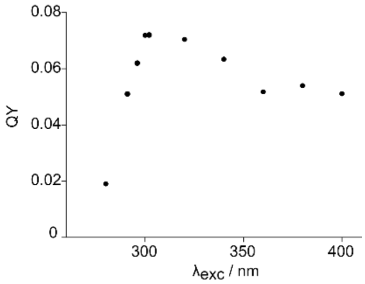

Figure S2. *RR*-CNDs-**1**. Luminescence QY measured upon changing the excitation wavelength in water.

B3. Characterization of *SS*-CNDs-1

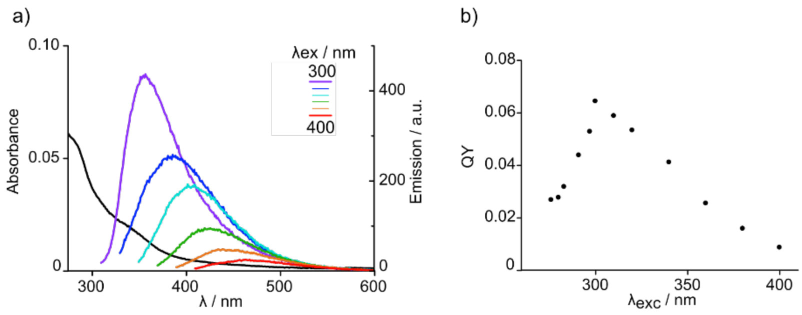

Figure S3. *SS*-CNDs-**1**. a) UV-Vis absorption and emission spectra recorded at different excitation wavelengths in water. b) Luminescence quantum yield upon changing the excitation wavelength in water.

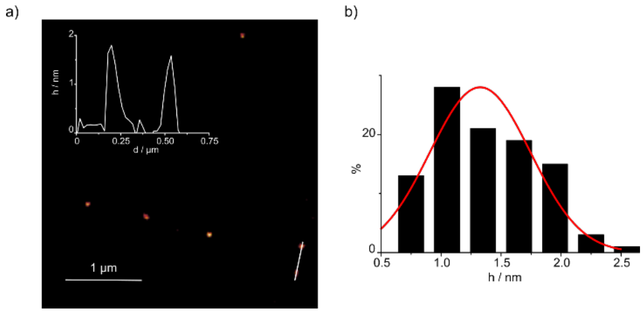

Figure S4. *SS*-CNDs-**1**. a) Tapping mode AFM of sample deposited on a mica substrate from drop cast of an aqueous solution; inset is the height profile along the white line. b) Size histogram of AFM height data, with distribution fit (red curve) based on a Gaussian distribution.

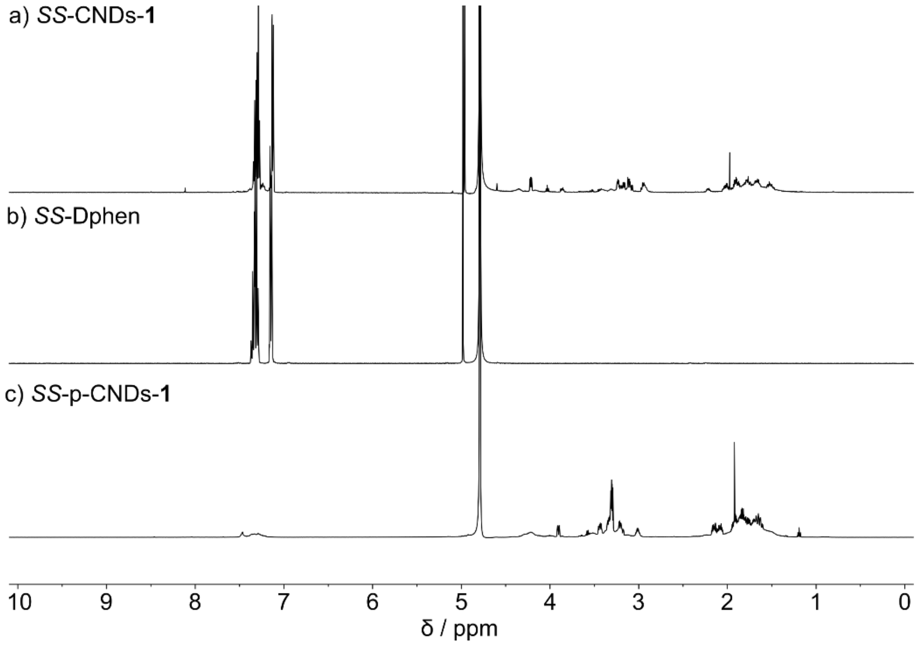

Figure S5. ^{1}H-NMR spectra (D_2O/TFA 3% v/v, 400 MHz, r.t.) of a) *SS*-CNDs-**1**, b) *SS*-Dphen, c) *SS*-p-CNDs-**1**.

B4. Characterization of *RR*-p-CNDs-1

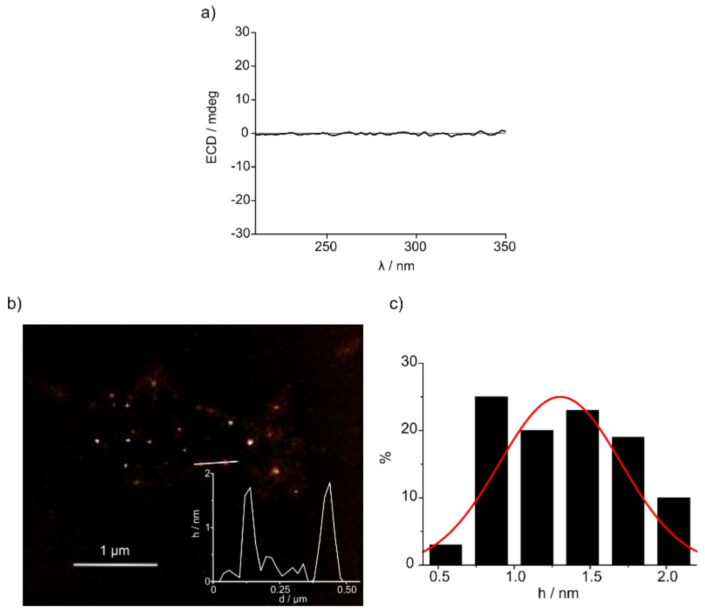

Figure S6. *RR*-p-CNDs-**1**. a) ECD spectrum recorded in water. b) Tapping mode AFM of sample deposited on a mica substrate from drop cast of an aqueous solution; inset is the height profile along the white line. c) Size histogram of AFM height data, with distribution fit (red curve) based on a Gaussian distribution.

B5. Characterization of *RR*-Dphen

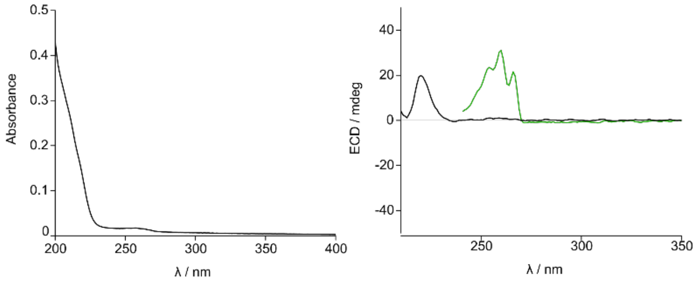

Figure S7. *RR*-Dphen. a) UV-Vis absorption spectrum recorded in water. b) ECD spectrum recorded in water; black line absorbance 0.87 at 220 nm, green line absorbance 0.83 at 260 nm.

C. CNDs-2

C1. Synthesis

CNDs-**2** were synthesized by following a literature procedure.[2] Briefly, *o*-phenylendiamine (*o*-PDA, 0.375 g), *L*-Tryptophan (Trp, 0.528 g), Milli-Q water (30 mL) and H_2SO_4 (1500 uL) were added to a 50 mL autoclave and heated at 160 °C for 7 h. After the reactors were cooled down naturally to room temperature, the solution was filtered with a filter membrane (0.1 µm) and dialyzed (cut off 500-1000 Da) for four hours. The aqueous solution was then lyophilized to obtain CNDs-**2** (153 mg). The characterization data of CNDs-**2** are in agreement with those reported in ref [2].

C2. Characterization

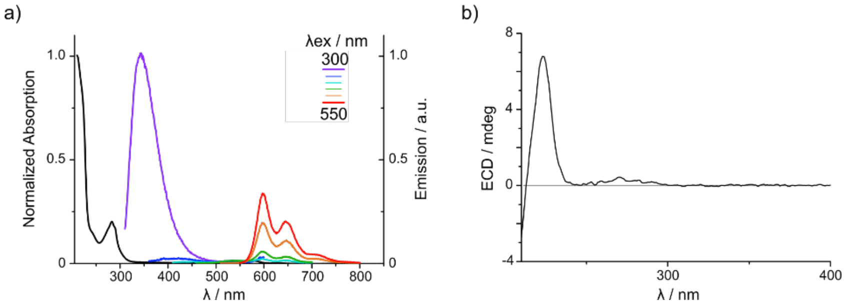

Figure S8. CNDs-**2**. a) UV-Vis absorption and emission spectra recorded at different excitation wavelengths in ethanol. b) ECD spectrum recorded in ethanol with an absorbance of 0.9 at 225 nm.

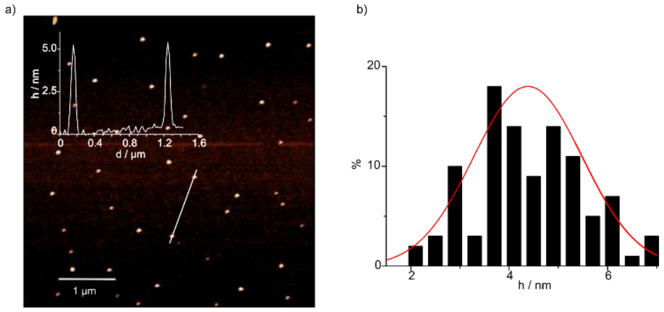

Figure S9. CNDs-**2**. a) Tapping mode AFM of sample deposited on a mica substrate from drop cast of methanol solution; inset is the height profile along the white line. b) Size histogram of AFM height data, with distribution fit (red curve) based on a Gaussian distribution.

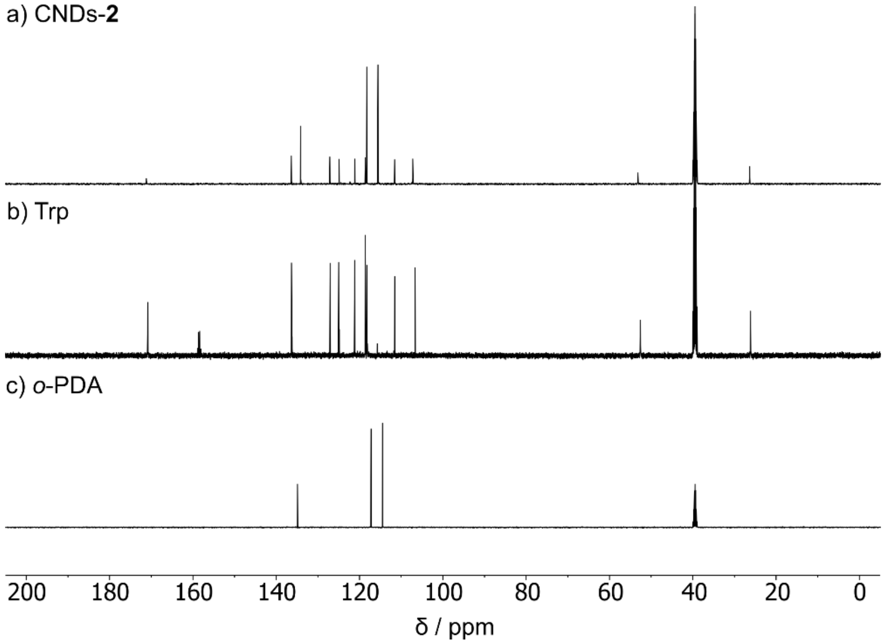

Figure S10. ^{13}C-NMR spectra (DMSO-d_6, 400 MHz, r.t.) of CNDs-**2,** Trp with 1 eq. of TFA-d, and *o*-PDA.

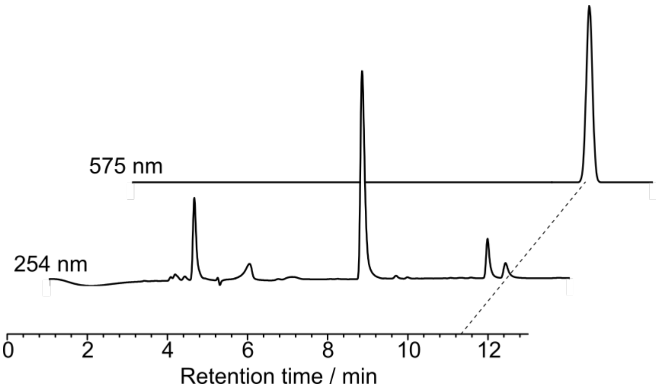

Figure S11. CNDs-**2**. Analytical C8-HPLC traces monitored at 254 and 575 nm.
Signal in the trace at 254 nm observed at ca. 5 min arise from *N,N*-dimethylformamide used as injected solvent, as confirmed by a control experiment.

C3. Fraction #1

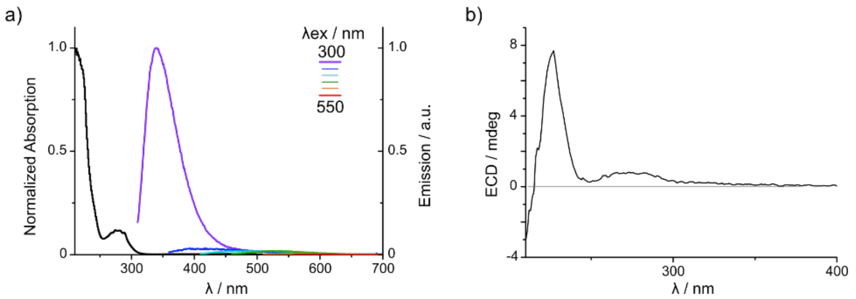

Figure S12. Fraction #1 isolated via semipreparative C18-HPLC separation. a) UV-Vis absorption and emission spectra recorded at different excitation wavelengths in ethanol. b) ECD spectrum recorded in ethanol with an absorbance of 1.5 at 225 nm.

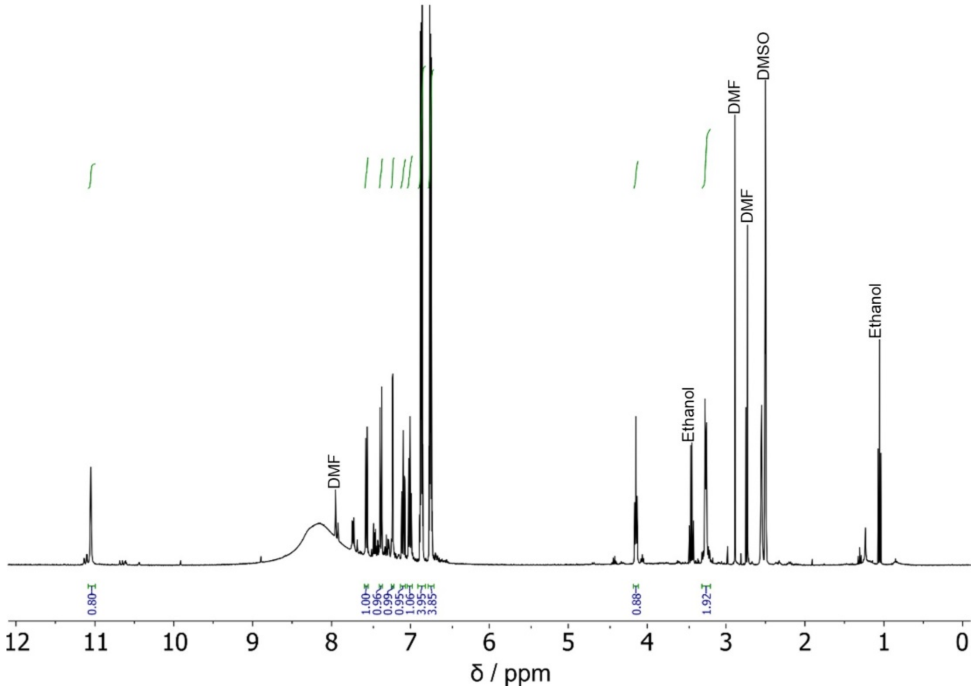

Figure S13. Fraction #1 isolated via semipreparative C18-HPLC separation. ^{1}H-NMR spectrum (DMSO-d$_6$, 400 MHz, r.t.).[a]

[a] The peaks observed in the ^{1}H-NMR spectrum besides *o*-PDA are ascribed to Trp, which could not be entirely separated under the employed semipreparative HPLC conditions. An additional analytical HPLC separation of fraction #1 under the same conditions affords *o*-PDA and Trp as cleanly separated peaks.

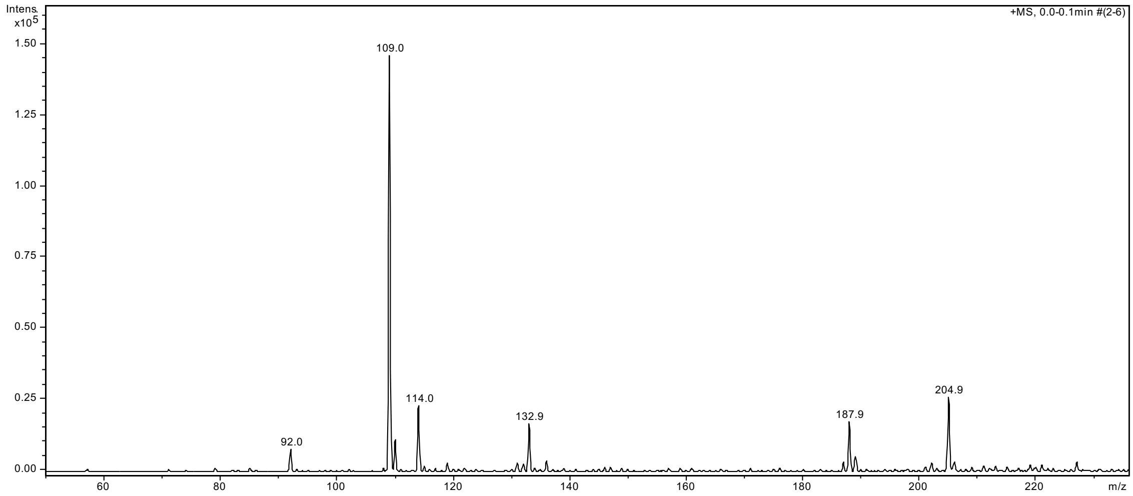

Figure S14. Fraction #1 isolated via semipreparative C18-HPLC separation. LRMS spectrum.

C4. Fraction #2

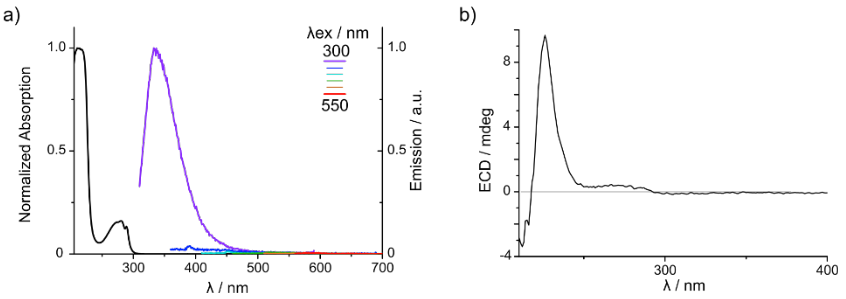

Figure S15. Fraction #2 isolated via semipreparative C18-HPLC separation. a) UV-Vis absorption and emission spectra recorded at different excitation wavelengths in ethanol. b) ECD spectrum recorded in ethanol with an absorbance of 1.5 at 225 nm.

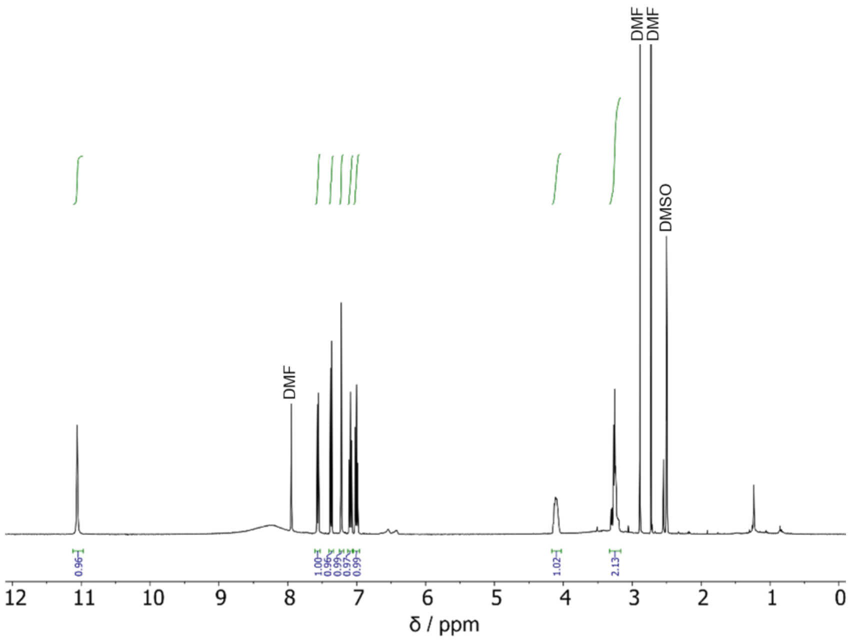

Figure S16. Fraction #2 isolated via semipreparative C18-HPLC separation. ^{1}H-NMR spectrum (DMSO-d$_6$, 400 MHz, r.t.).

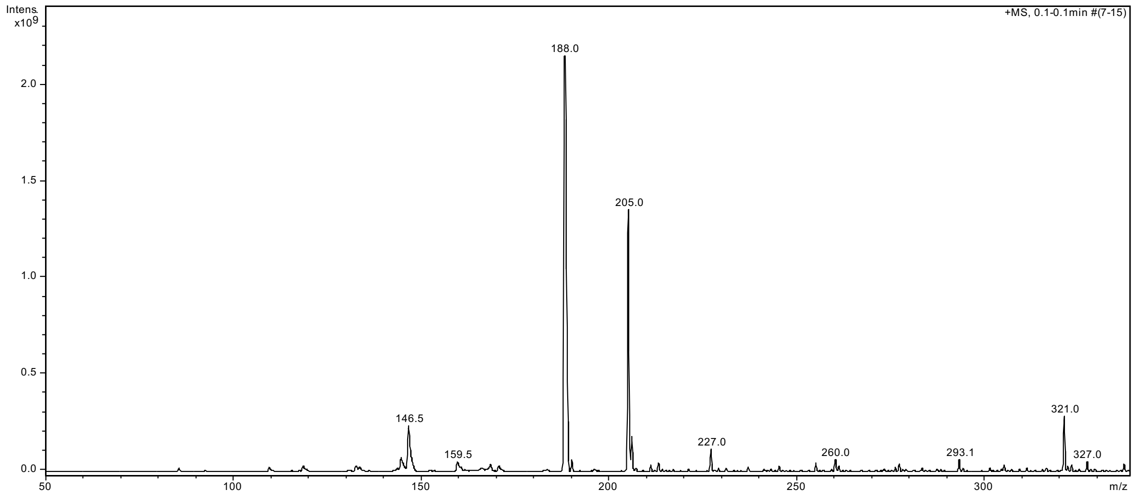

Figure S17. Fraction #2 isolated via semipreparative C18-HPLC separation. LRMS spectrum.

C5. Fraction #3

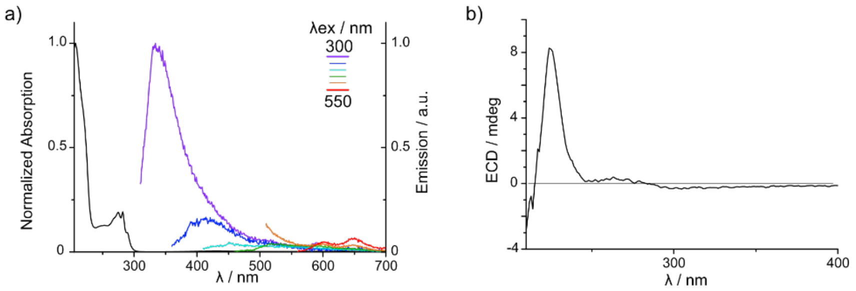

Figure S18. Fraction #3 isolated via semipreparative C18-HPLC separation. a) UV-Vis absorption and emission spectra recorded at different excitation wavelengths in ethanol. b) ECD spectrum recorded in ethanol with an absorbance of 1.5 at 225 nm.

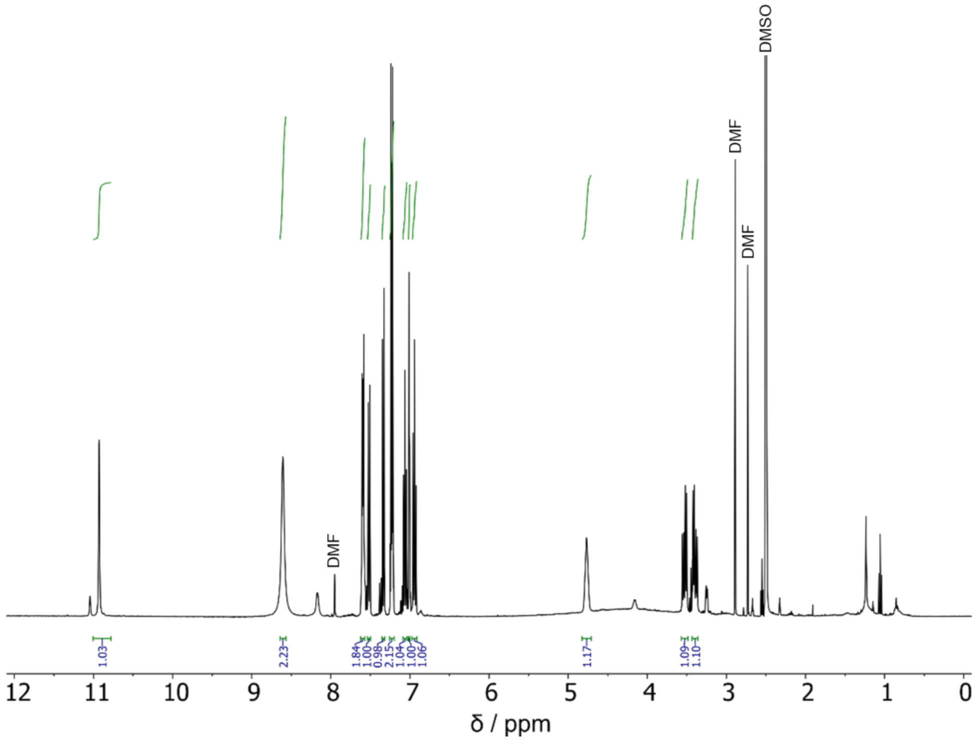

Figure S19. Fraction #3 isolated via semipreparative C18-HPLC separation. ^{1}H-NMR spectrum (DMSO-d$_6$, 400 MHz, r.t.).

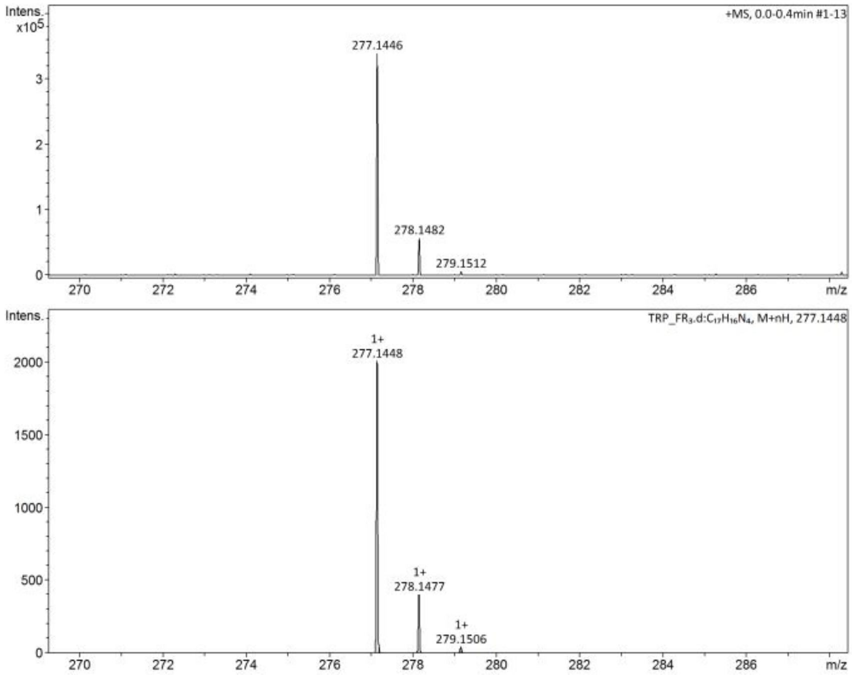

Figure S20. Fraction #3 isolated via semipreparative C18-HPLC separation. HRMS; experimental spectra (top), simulated spectra (bottom).

C6. α-(2-Benzimidazolyl)-β-(3-indolyl)-ethylamine (3)

C6.1. Synthesis

α-(2-Benzimidazolyl)-β-(3-indolyl)-ethylamine was synthesized by following a literature procedure.[3] Typically, a flame-dried 50 mL flask was charged with Trp (4 mmol, 817 mg), *o*-PDA (5 mmol, 541 mg) and $SnCl_2$ (0.7 mmol, 133 mg). Then, the reactants were heated to 170 °C and the reaction was carried out for 5 hours. The crude product was purified by semipreparative C18-HPLC. The characterization data of **3** are in agreement with those reported in ref [3].

C6.2. Characterization

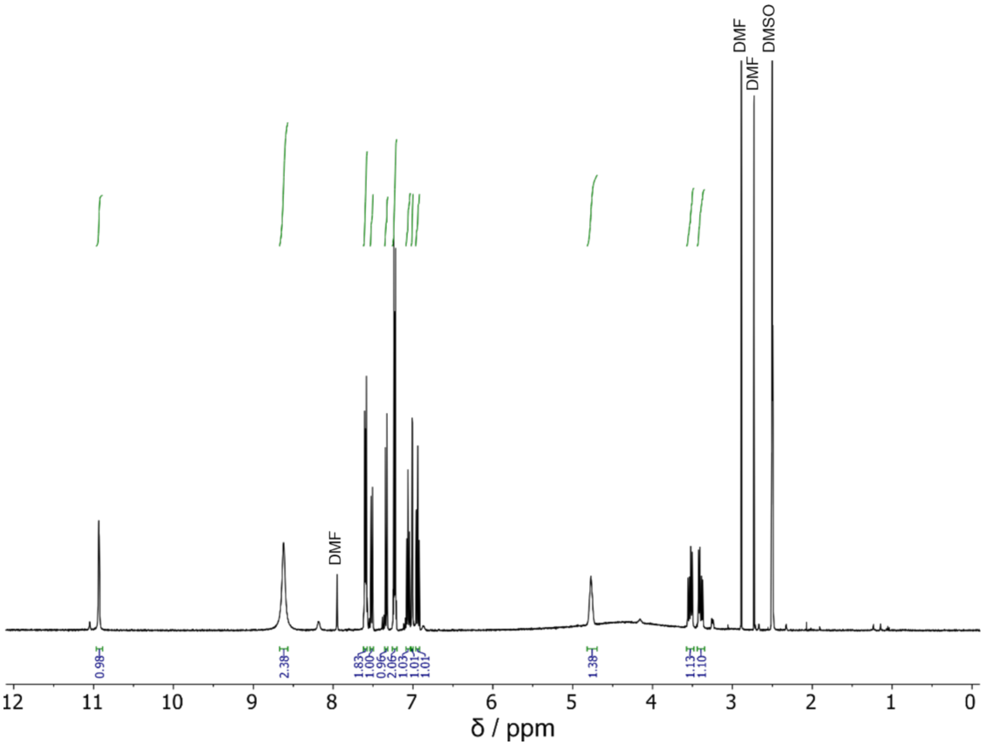

Figure S21. Compound **3**.^{1}H-NMR spectrum (DMSO-d_6, 400 MHz, r.t.).

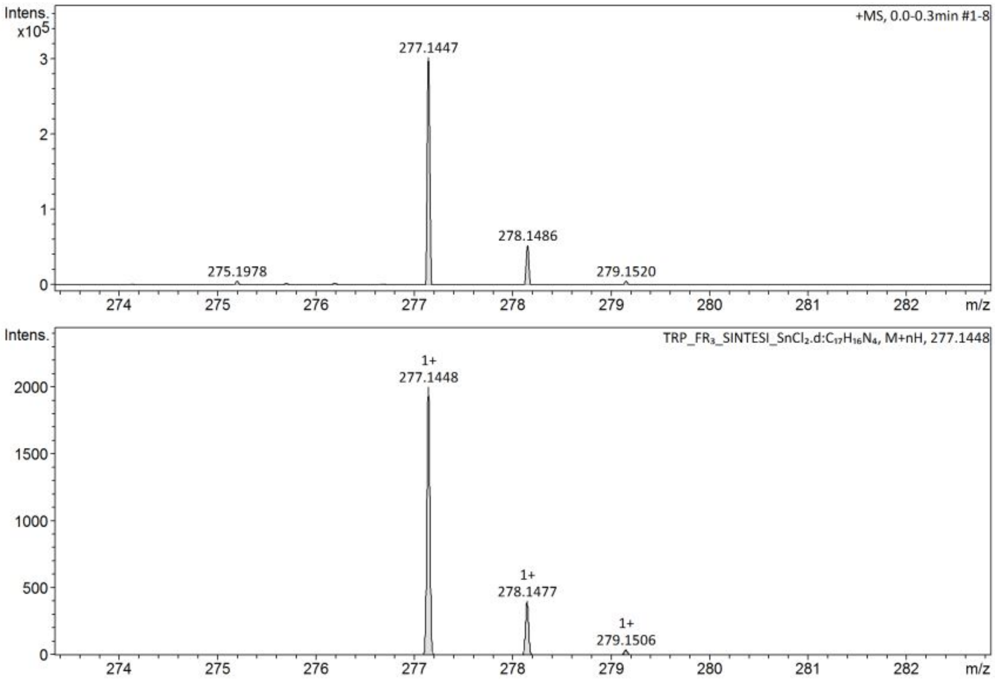

Figure S22. Compound **3.** HRMS, experimental spectra (top), simulated spectra (bottom).

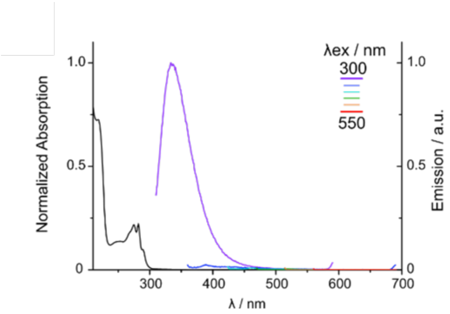

Figure S23. Compound **3**. UV-Vis absorption and emission spectra recorded at different excitation wavelengths in ethanol.

C7. Fraction #4

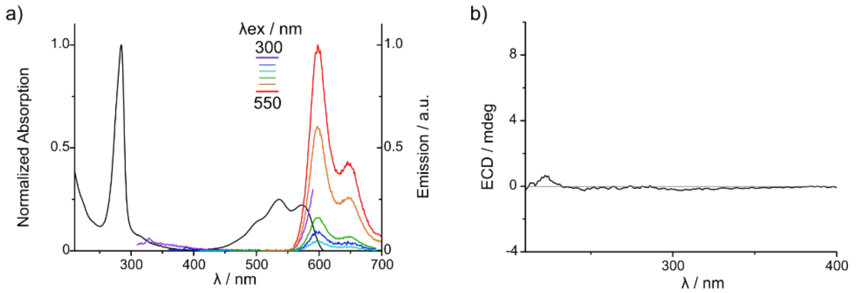

Figure S24. Fraction #4 isolated via semipreparative C18-HPLC separation. a) UV-Vis absorption and emission spectra recorded at different excitation wavelengths in ethanol. b) ECD spectrum recorded in ethanol with an absorbance of 0.3 at 225 nm.

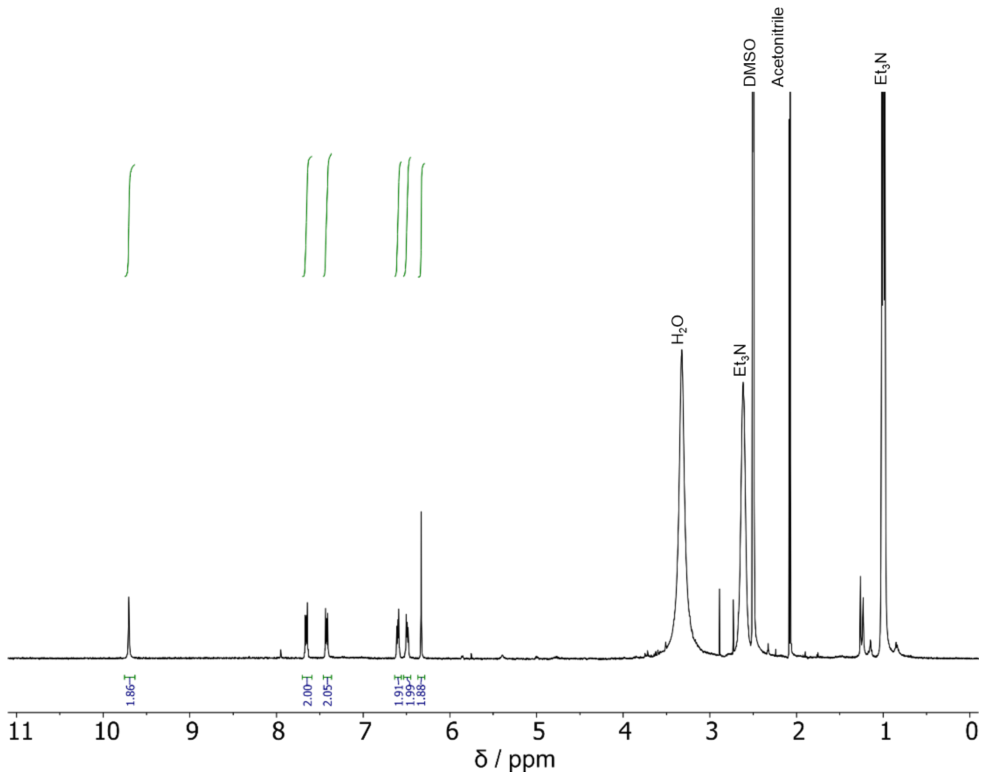

Figure S25. Fraction #4 isolated via semipreparative C18-HPLC separation. ^{1}H-NMR spectrum (DMSO-d$_6$/triethylamine, 400 MHz, r.t.).

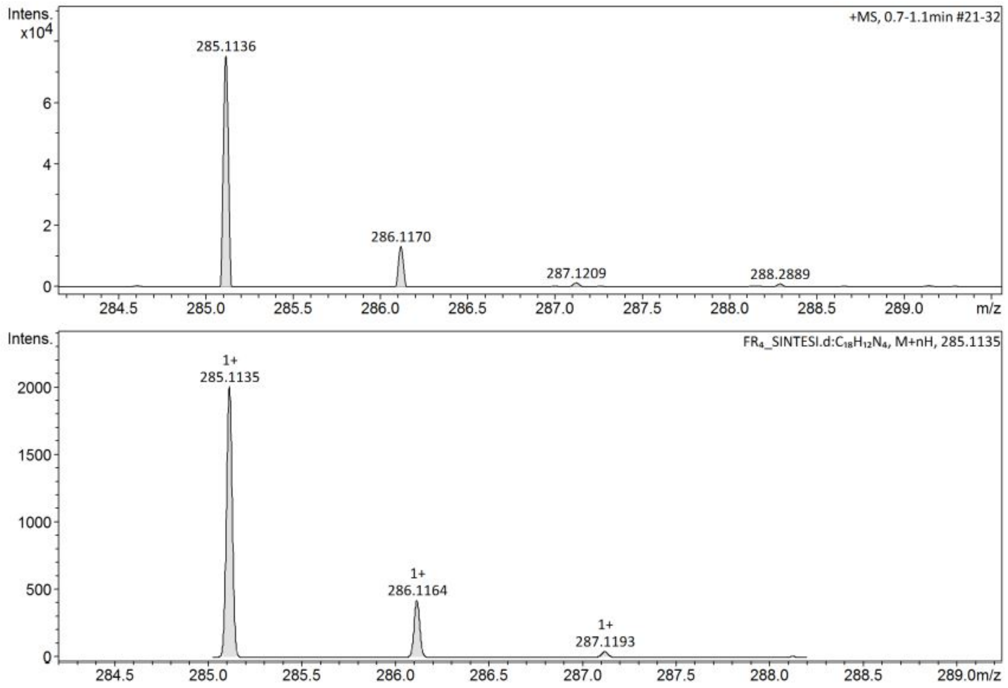

Figure S26. Fraction #4 isolated via semipreparative C18-HPLC separation. HRMS; experimental spectra (top), simulated spectra (bottom).

C8. 5,14-dihydro-5,7,12,14-tetraazapentacene (4)

C8.1. Synthesis

5,14-dihydro-5,7,12,14-tetraazapentacene was synthesized by following a literature procedure.[4] Typically, 2,5-dihydroxy-1,4-benzoquinone (3 mmol, 0.42 g) and *o*-PDA (15 mmol, 1.62 g) were mixed and heated to 160 °C for 5 hours under Argon atmosphere. The crude product was purified by semipreparative C18-HPLC. The characterization data of **4** are in agreement with those reported in ref [4].

C8.2. Characterization

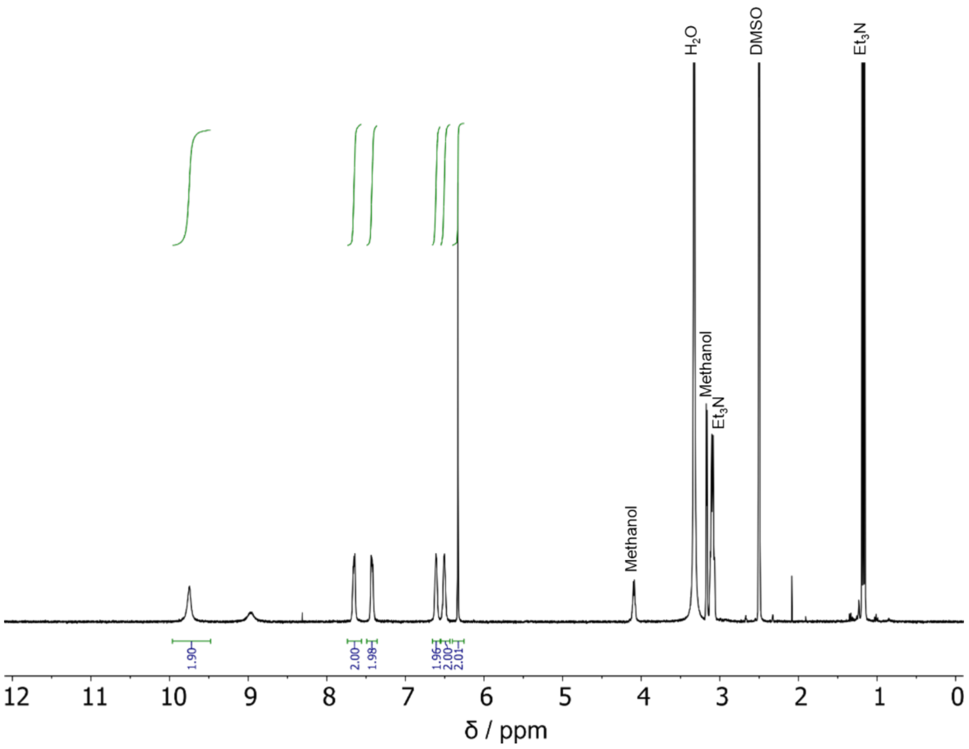

Figure S27. Compound **4**.^{1}H-NMR spectrum (DMSO-d_6/triethylamine, 400 MHz, r.t.).

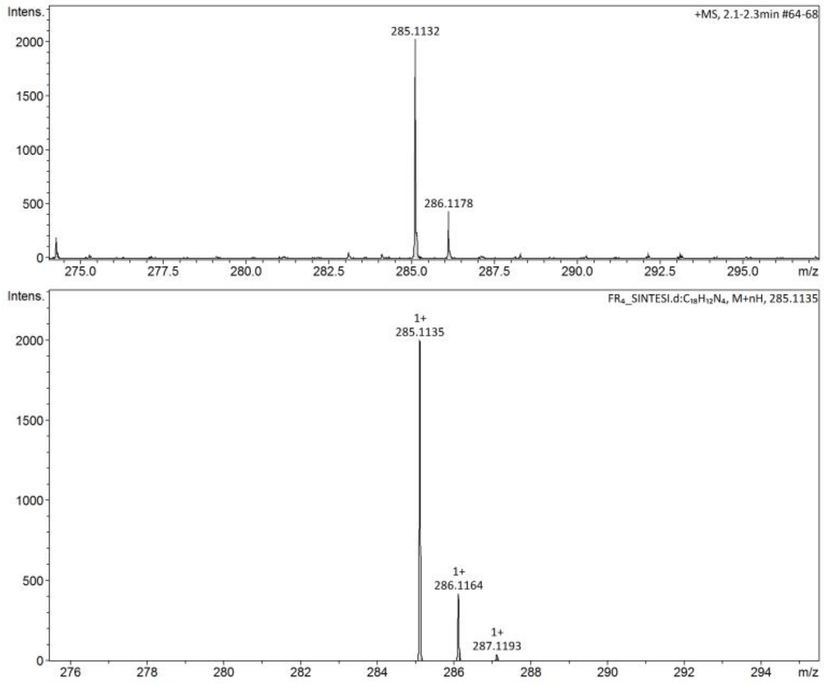

Figure S28. Compound **4**. HRMS, experimental spectra (top), simulated spectra (bottom).

C9. Characterization of *L*-Tryptophan

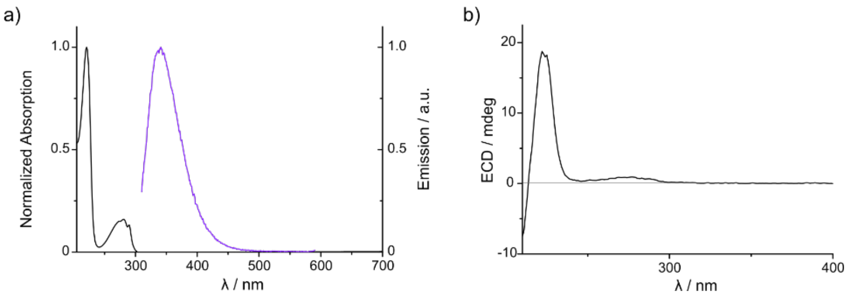

Figure S29. *L*-Tryptophan. a) UV-Vis absorption and emission spectra recorded in ethanol. b) ECD spectrum recorded in ethanol with an absorbance of 2 at 225 nm.

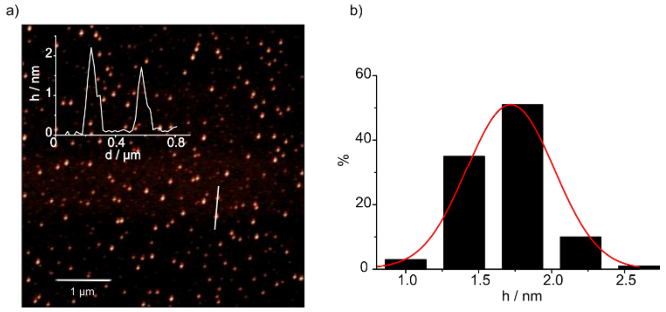

Figure S30. *L*-Tryptophan. a) Tapping mode AFM of sample deposited on a mica substrate from drop cast of a methanol solution; inset is the height profile along the white line. b) Size histogram of AFM height data, with distribution fit (red curve) based on a Gaussian distribution.

D. References

[1] F. Arcudi, L. Đorđević, M. Prato, *Angew. Chem. Int. Ed.* **2016**, *55*, 2107–2112.
[2] Y. Ru, L. Sui, H. Song, X. Liu, Z. Tang, S. Q. Zang, B. Yang, S. Lu, *Angew. Chem. Int. Ed.* **2021**, *60*, 14091–14099.
[3] R. H. Chen, J. F. Xiong, P. Peng, G. Z. Mo, X. S. Tang, Z. Y. Wang, X. F. Wang, *Asian J. Chem.* **2014**, *26*, 926–932.
[4] Q. Tang, J. Liu, H. S. Chan, Q. Miao, *Chem. Eur. J* **2009**, *15*, 3965–3969.
